# A follow up study on the efficacy of metadoxine in the treatment of alcohol dependence

**DOI:** 10.1186/1747-597X-1-35

**Published:** 2006-12-18

**Authors:** Irene Guerrini, Claudio Gentili, Gloria Nelli, Mario Guazzelli

**Affiliations:** 1 Bexley Substance Misuse Service, South London and Mausdley NHS Trust, London, UK; 2 Molecular Psychiatry Laboratory, Windeyer Institute of Medical Sciences, Department of Mental Health Sciences, Royal Free and University College London, London Medical School, 46 Cleveland Street, London W1T 4JF ,UK; 3 Department of Psychiatry, Neurobiology, Pharmacology and Biotechnologies, University of Pisa, Via Roma 67, 56124 Pisa, Italy

## Abstract

**Background:**

We carried out a three months follow-up study on the efficacy of metadoxine in a cohort of alcoholics admitted to the Alcohol misuse Long-term Treatment (ALT) Unit – University of Pisa (Italy). We analyzed the clinical data, psychometric tests and blood tests of 160 alcoholics on admission and after 3 months of treatment. We compared 58 pts treated with metadoxine (MET) with 102 pts who did not receive (NULL) any drug as an adjunct to the psycho-educational interventions provided by the ALT Unit.

**Results:**

At follow-up, the patients in treatment with metadoxine showed a significant improvement in the rate of complete abstinence (44.8% vs. 21.6%; chi square: 8.45, df = 1, p < 0.0037). Furthermore, the number of drop-outs at three months of treatment was also significantly lower in the MET than in the NULL group (17% vs. 57%; chi square of 23.22, df = 1, p < 0.001).

**Conclusion:**

Our findings support the use of metadoxine in the management of alcohol dependence. However, randomized clinical trials are necessary to confirm and replicate them. This study raises the importance of identifying new pharmacological compounds effective on the outcome of alcoholism in order to help patients to best adhere to treatment programs and to prevent the development of mental and physical complications due to chronic and heavy use of alcohol.

## Background

Alcohol misuse is a common problem in the general population all over the world. Recent surveys reported that alcoholism affects almost 10–15% of the general population in US and 1–5% in Europe [1-5]. There is a general agreement in the scientific community that heavy drinking is largely underestimated by clinicians and that alcohol misusers are often not detected unless and until they develop severe alcohol related pathologies [6,7]. According to Stickel and co-workers (2003) in Europe more than 45 million individuals showed signs of alcohol-related damage, mainly cognitive deficits, liver disease, and myopathies [8-11]. The importance of the nutritional status in the development of alcohol-related organ damage has been postulated by several authors [8,12,13].

A reduction in the blood levels of micro and macronutrients, mainly B-complex vitamins, can impair the cognitive functions and therefore compromise the recovery of alcoholics [14]. In order to prevent brain damage and consequential cognitive impairment, the guidelines for the Accident & Emergency departments in United Kingdom recommend the use of parenteral B vitamins for patients showing evidence of chronic alcohol misuse and suspected of having a poor diet [15]. The B-complex vitamins (i.e. thiamine, pyridoxine etc) are extremely important for the homeostasis of the brain, being key factors in the cell metabolism and trafficking, in the energy production pathways and in the DNA synthesis [16].

Therefore, it is important in the treatment of alcohol misuse to consider an integrated approach that includes pharmacological and psychological intervention, social support and nutritional supplementation.

Several studies have proposed the use of metadoxine in the treatment of acute and chronic alcohol misusers [17-21]. Metadoxine is an ion-pair between pyrrolidon carboxilate (PCA) and pyridoxine (vit. B6) with the two compounds linked in a single product by salification. This process synergistically increases its pharmacological activity [17]. In animal studies metadoxine increases the plasma and urinary excretion of ethanol, inhibits the increased production of fatty acid esters in the liver during chronic alcohol intake, reduces oxidative stress and prevents glutathione depletion in the hepatic tissues [17]. In the brain metadoxine increases the level of GABA and acetylcholine in the frontoparietal cortex of guinea pigs [22]. In mice it increases the level of dopamine in the striatum[23,24].

In human studies, it has also been postulated that metadoxine is effective in maintaining abstinence, in decreasing the craving for alcohol and in improving the cognitive function mainly short-term memory [17,19,20]. The improvement of the short-term memory can be related to the effect of this compound on the cholinergic and gabaergic system reported by Antonelli and coworkers in guinea pigs [22].

Clinical trials showed that in acutely intoxicated patients metadoxine reduces the ethanol blood levels and increases the urinary clearance of ethanol and its metabolites [21]. Metadoxine seems to be effective in the recovery of fatty liver and in improving the laboratory blood tests [18] and, as shown by Shpilenya and coworkers (2002), it seems to ameliorate the clinical and behavioural symptoms during alcohol intoxication [21]. Caballeria and co-workers (1998) reported in a double-blind randomized multicentre study that metadoxine significantly improves the liver enzymes and reduces the rate of steatosis after just a month of treatment [18].

We carried out a follow-up study on the efficacy of metadoxine in a cohort of alcoholics admitted to the Alcohol misuse Long-term Treatment (ALT) Unit at the Institute of Psychiatry University of Pisa (Italy). The purpose of this study is to test the potential benefits of using metadoxine in the management of Alcohol Dependence.

## Results

The 160 patients were 134 males and 26 females (mean age 44 yrs; SD = 11.) At admission at ALT Unit all the patients were completely detoxified and totally abstinent from alcohol. The fifty-eight patients in treatment with metadoxine received a daily dose of metadoxine of 1000 mg, orally administered three times a day.

The clinical characteristics of the samples are shown in Table 1. The alcohol intake refers to the amount consumed before admission to the ALT Unit. The intake is calculated in grams of alcohol according to Italian standards (see Guerrini et al., 2006 as a reference)[5]. The Family History (FH) for Alcohol Dependence was evaluated by two independent clinicians, interviewing the patient and one of his/her first degree relatives. The Age of onset refers to the age in which the individuals started having signs/symptoms of Alcohol Dependence.

**Table 1:** Clinical characteristics of the patients recruited in the study. The alcohol intake refers to the amount consumed (gr/day) before admission to the ALT Unit.

	**Total sample**	**MET**	**NULL**
**Samples size (no.)**	160	58	102
**Age (yrs.)**	44 (SD: 11)	45 (SD: 11)	44 (SD: 12)
**Gender**			
**Males**	184	51	83
**Females**	26	7	19
FH for Alcohol Dependence*			
**Negative**	55	17	38
**Positive**	105 (71%)	41 (63%)	64 (64%)
Alcohol intake prior to admission to the ALT Unit**	189 (SD:62)	175 (SD: 70)	201 (SD: 82)
**Age of onset of Alcohol Dependence**	27 (SD:11)	26 (SD:10)	27 (SD:12)

(*) Note: FH refers to Family History.

(**) Note: The intake is calculated in grams of alcohol according to Italian standards (see Guerrini et al., 2006 as a reference).

Table 2 shows the mean scores of the rating scales at admission and after three months of treatment and the analysis of the covariance. Only the MAST score was significantly changed between the admission and the follow up evaluation (One-way ANCOVA, F = 9.76, df = 1, df error = 9, p < 0.01).

**Table 2:** Mean values of the rating scales according to treatment and time points and ANCOVA p-values. SD = Standard Deviation.

	MET		NULL		ANCOVA analysis(*)
	Baseline	Follow-up	Baseline	Follow-up	

MALT1	14 (SD:5)	8 (SD:7)	12 (SD:5)	12 (SD:11)	P < 0.579
MALT2	16 (SD:5)	10 (SD:8)	14 (SD:5)	11 (SD:8)	P < 0.08
MAST	25 (SD:8)	18 (SD:2)	18 (SD:1)	14 (SD:11)	P < 0.01
HRSA	10 (SD:7)	8 (SD:8)	13 (SD:9)	7 (SD:7)	P < 0.8
HRSD	10 (SD:6)	7 (SD:7)	12 (SD:7)	9 (SD:8)	P < 0.47

(*) Note: p-values are from an F-test with 1, 9 df.

Table 3 shows the means and ANCOVA analysis of the blood tests of the two groups (MET and NULL) at admission and at three months follow-up.

**Table 3:** Mean values of the rating scales according to treatment and time points and ANCOVA p-values. SD = Standard Deviation.

	MET		NULL		ANCOVA analysis(*)
	Baseline	Follow-up	Baseline	Follow-up	

γ GT	290 (SD:386)	165 (SD:56)	190 (SD:309)	71 (SD:74)	P < 0.77
GOT	71 (SD:75)	37 (SD:21)	51 (SD:39)	38 (SD:21)	P < 0.53
GPT	62 (SD:72)	32 (SD:25)	44 (SD:40)	44 (SD:26)	P < 0.54
MCV	97 (SD:10)	92 (SD:7)	97 (SD:8)	96 (SD:8)	P < 0.89

(*) Note: p-values are from an F-test with 1, 9 df.

In terms of compliance, the cases in the MET group showed a significantly lower number of dropouts compared to the NULL group (17% vs. 57%; chi square of 23.22, df = 1, p < 0.001), data shown in Table 4.

**Table 4:** Clinical outcome of the patients recruited in the study at three months follow-up.

	MET	NULL	Chi^2 ^analysis
No. patients	58	102	
Drop-outs	10	59	23.22, df = 1; p < 0.0001
Complete abstinence	26	22	8.45, df = 1; p < 0.0037

Furthermore, the group treated with metadoxine showed a better outcome in terms of complete abstinence after three months of treatment (44.8% vs. 21.6%; chi square: 8.45, df = 1, p < 0.0037), data showed in Table 4.

## Conclusion

The efficacy of metadoxine in the management of alcoholism has been postulated by several authors [17,19-21]. The use of this compound has been analysed in several pathological conditions from the acute alcohol intoxication to the liver pathology in chronic alcoholism [17] but very few follow-up studies explored its role in the medium and long-term treatment of Alcohol Dependence. Our follow-up study covered a period of only three months and was biased by the fact that the cases were not assigned completely at random to each experimental condition.

Our findings indicate that the group of patients treated with metadoxine shows a better three months outcome both in terms of drop-out rate and complete abstinence. These results are in accordance to the data reported by Rizzo and co-workers (1993) that showed a better improvement of the patients treated with metadoxine in terms of abstinence maintenance at least in the short term [19].

Regarding the psychometric rating scales score for alcoholism only the MAST test showed a statistically different improvement in the metadoxine treated group suggesting a global improvement of the Alcohol Dependence.

All these findings suggest a possible effect of the metadoxine on the central nervous system. Several animal studies postulate the role of this compound in the dopamine release and in the GABA pathway. An anti-craving effect has been suggested to explain the ability of this compound to induce alcohol abstinence and to improve clinical symptoms [17].

Both the pyrrolidon carboxilate (PCA) and the pyridoxol are also important molecules involved in the energy metabolism and in maintaining the homeostasis of the brain. Wei et coworkers (1999) showed that serious cognitive deficit occurs in vitamin B(6)-deficient animals [25]. Nutritional deficiency is an important element in the pathogenesis of cognitive impairment and consequently affects the patients' compliance to the treatment. Furthermore PCA has been shown to have antioxidant effects and seems to reduce the aging process in the brain [26]. Our study suggests the importance of evaluating the role of compounds such as micro and macronutrients (piridoxine, thiamine, folic acid, magnesium etc) in the treatment of alcohol misuse. For example, the role of thiamine in the prevention of cognitive deficits and severe brain damage in alcoholics is well accepted by the scientific community. Pharmacological compounds that show an effect on the rate of complete abstinence are of extreme importance in the treatment of alcohol dependence, a chronic relapsing disorder by definition. In fact, in the literature it is quite well known that the first three months of treatment are very critical in terms of relapsing into drinking and dropping out from services. It is important to consider this phenomenon when planning treatment policy and therefore the early introduction of relapse prevention measures, both psychological and pharmacological, becomes an essential part of the treatment.

Despite all the limitations of our study (small sample size and non randomisation) the findings support the use of metadoxine in the management of alcohol dependence. Our study raises the necessity to investigate the long-term efficacy of metadoxine in prospective, randomized, controlled trials in large samples.

## Methods

We included in the study 160 alcoholics admitted to the Alcohol misuse Long-term Treatment (ALT) Unit at the Institute of Psychiatry University of Pisa (Italy) in a period of a year. All the patients were discharged by different detoxification units after 15–20 days of treatment and admitted to the ALT Unit in order to start a long-term treatment program, which included psychiatric counselling, self-help group therapy (Club of Alcoholics in Treatment) and pharmacological treatment. We analyzed the clinical data, psychometric tests and blood tests at the admission in the ALT Unit and after three months of rehabilitative treatment.

Fifty eight alcoholics were in treatment with metadoxine (MET) as an adjunct to the rehabilitative interventions and 102 patients (NULL) were drugs free. The assignment to the metadoxine treatment was not randomised but was left to the clinical judgement at admission in the ALT Unit.

The patients included in the study were all Alcohol Dependent according to DSM III-R criteria and without any severe medical conditions. At admission in the ALT Unit the patients received a baseline evaluation that included: psychiatric interview, blood tests and psychometric rating scales specific for Alcohol Dependence (Munich Alcoholism Test 1 and 2; Michigan Alcoholism Screening Test) and for anxiety and depression (Hamilton Rating Scale for Anxiety- HRSA and for Depression-HRSD). According to the therapeutic protocol of the ALT Unit after three months of treatment all the patients were clinically re-evaluated, the blood tests were repeated and the rating scales were administered again. The psychometric rating scales were used in order to evaluate changing in the drinking habits and in the depressive and anxiety symptoms induced by the alcohol misuse. All the scales used in the present studies were validated in different populations and used in several studies [27,28]. At this stage the first evaluation of the treatment outcome was carried out. Several parameters were taken into account: 1) alcohol intake was estimated according to the patients self-reporting, the weekly report of the self-help groups and interviewing close relatives; 2) number of relapses; 3) length of the period of complete abstinence between two consecutive relapses; 4) drop-out rate.

### Statistics

To maximise precision of estimates, analysis of covariance (ANCOVA) was performed on the rating scales scores and on the blood tests at baseline and at three months of treatment, using the follow-up data as the dependent measure, the different treatment group as the independent factor and the baseline data as the covariate. The outcomes analysed were the number of dropouts and the complete abstinence rate calculated as the number of individuals who completely abstain for all the three months period. The statistical comparison was carried out using a chi square test with 1 degree of freedom. Statistical analysis was performed using the SPSS/PC+ package.

## Competing interests

The author(s) declare that they have no competing interests.

## Authors' contributions

IG carried out the project recruiting the patients and collecting all the clinical data and drafted the manuscript. CG participated in the design of the study and performed the statistical analysis. GN participated in the clinical assessment. MG conceived the study, and participated in its design and coordination and helped to draft the manuscript. All authors read and approved the final manuscript.
